# Closed-Chain Inverse Dynamics for the Biomechanical Analysis of Manual Material Handling Tasks through a Deep Learning Assisted Wearable Sensor Network

**DOI:** 10.3390/s23135885

**Published:** 2023-06-25

**Authors:** Riccardo Bezzini, Luca Crosato, Massimo Teppati Losè, Carlo Alberto Avizzano, Massimo Bergamasco, Alessandro Filippeschi

**Affiliations:** 1 Institute of Mechanical Intelligence, Scuola Superiore Sant’Anna, 56127 Pisa, Italy; r.bezzini@santannapisa.it (R.B.); m.teppatilose@santannapisa.it (M.T.L.); c.avizzano@santannapisa.it (C.A.A.); m.bergamasco@santannapisa.it (M.B.); 2 Department of Computer and Information Sciences, Northumbria University, Newcastle upon Tyne NE1 8ST, UK; luca.crosato@northumbria.ac.uk; 3 Department of Excellence in Robotics and AI, Scuola Superiore Sant’Anna, 56127 Pisa, Italy

**Keywords:** biomechanics, ergonomics, load estimation, wearable sensor networks, inertial measurement units

## Abstract

Despite the automatization of many industrial and logistics processes, human workers are still often involved in the manual handling of loads. These activities lead to many work-related disorders that reduce the quality of life and the productivity of aged workers. A biomechanical analysis of such activities is the basis for a detailed estimation of the biomechanical overload, thus enabling focused prevention actions. Thanks to wearable sensor networks, it is now possible to analyze human biomechanics by an inverse dynamics approach in ecological conditions. The purposes of this study are the conceptualization, formulation, and implementation of a deep learning-assisted fully wearable sensor system for an online evaluation of the biomechanical effort that an operator exerts during a manual material handling task. In this paper, we show a novel, computationally efficient algorithm, implemented in ROS, to analyze the biomechanics of the human musculoskeletal systems by an inverse dynamics approach. We also propose a method for estimating the load and its distribution, relying on an egocentric camera and deep learning-based object recognition. This method is suitable for objects of known weight, as is often the case in logistics. Kinematic data, along with foot contact information, are provided by a fully wearable sensor network composed of inertial measurement units. The results show good accuracy and robustness of the system for object detection and grasp recognition, thus providing reliable load estimation for a high-impact field such as logistics. The outcome of the biomechanical analysis is consistent with the literature. However, improvements in gait segmentation are necessary to reduce discontinuities in the estimated lower limb articular wrenches.

## 1. Introduction

Manual Material Handling (MMH) is still one of the most common human activities in logistics and industrial contexts, despite the enormous progress in automation. The aging of the workers’ population is exacerbating the impact of work-related musculoskeletal disorders (WMSDs) related to MMH on workers’ health (e.g., [1] in Europe). This situation negatively affects workers’ quality of life, productivity, and the costs for national healthcare systems. Prevention of WMSDs through mitigation of the biomechanical overload risk is the best strategy to cope with this problem.

Prevention actions include total or partial (e.g., collaborative robotics) automation of processes [2,3], exoskeletons for the human workers [4,5], and re-design of MMH tasks and tools. All these prevention actions need the evaluation of the ergonomic risk and can highly benefit from a biomechanical analysis of MMH tasks.

Standardized methods for the evaluation of the ergonomic risk related to MMH are reported in the ISO norms for the assessment of the risks correlated to posture (ISO 11226) and MMH (ISO 11228-1-2-3). These methods are observational and typically require many human kinematics variables for their computation. At the same time, they often demand only a coarse knowledge of the external loads to compute a score of the ergonomic risk.

Although ergonomic assessment is carried out traditionally by visual inspection of videos of the working activity, wearable sensing technologies, such as inertial measurement units and electromyography have been adopted for partial or complete automation of the evaluation process [6,7]. These technologies have allowed evaluators to use quantitative measurements of the kinematic variables, thus improving the accuracy and the repeatability of the evaluation.

In recent years, however, some ergonomists recognize the need to enrich these methods with information related to the actual loading of the musculoskeletal system [8], which can be obtained through a biomechanical analysis of MMH tasks based on models of the human musculoskeletal system. Beyond risk assessment, biomechanical models are useful tools for the design of prevention actions. They can serve for the definition of the specifications of assistive exoskeletons, for the organization of production lines that include collaborative robots, or be extended to the more general paradigm of Virtual Commissioning, which is promising in Computer-aided manufacturing to reduce production costs and failures, which are detrimental to companies’ reputation [9,10]. Therefore, the purposes of this study are the conceptualization, formulation, and implementation of a deep learning-assisted fully wearable sensor system for an online evaluation of the biomechanical efforts that an operator exerts during an MMH task.

Thanks to the aforementioned technologies nowadays available for motion tracking, human posture, and motion are assumed to be known in all these applications. Therefore, we focus on methods based on inverse dynamics. The models that have been proposed to cope with this problem can be split into two groups.

The first group exploits a detailed representation of the human musculoskeletal system. These models include almost all the human bones, properly connected by modeling the surface contacts among bones and considering muscles as actuation units with inertial, elastic, and damping properties as well as actuation functions. The resulting musculoskeletal models are inevitably mechanically redundant as several muscles span each joint, and different compositions of muscle forces can generate a net joint moment. The solution of the redundancy is often obtained using optimization functions (e.g., [11,12]) that, for example, may assume as a criterion the minimization of muscles tension/stress [13]. Another adequate resolution approach consists in exploiting muscular activation data (EMG-driven methods) [14,15,16]. These models require extensive information about human body properties and typically do not run in real time. For the implementation of such methods, a state-of-the-art, open-source framework is OpenSim [17], a modular platform that allows users to build models and solve them using the Simbody solver [18]. An example of a real-time application that exploits the OpenSim platform is the one proposed in [19]. However, here the authors do not take into account the possibility of multiple contact points different from the double support. Moreover, the mentioned work does not consider the presence of external loads, which is crucial in MMH.

Simpler methods, belonging to the second approach, do not include modeling of each muscle, but model the human body in a robotics-like fashion: each bone is a rigid link, which is connected to its neighbors through rotational, hinge, or ball joints, and is actuated by wrenches lumped at the joints [20,21,22]. This approach requires far less information about the human body to run a simulation hence allowing for online estimation of the human joint torques. Indeed, such a computationally convenient modeling method is widely used for real-time assessments of forces and torques to which an operator’s joints are subjected (e.g., [23,24]).

We consider robotics-like models the ideal compromise between the richness of the model and its simplicity. To support this statement, we report a study [25] that investigated the correlation between a standardized ergonomic risk assessment method (i.e., RULA [26]), and methods belonging to the first and the second type. Results show a better correlation of the second type model with the RULA method.

Therefore in this paper, we have adopted the second approach for three reasons. Firstly, we want to make it easy to set up the human model for pervasive use, e.g., in a workplace to assess ergonomic risks. Hence, we want the model to require as few parameters as possible. Secondly, we want our method to be suitable for real-time execution to provide timely feedback to workers for everyday use. Lastly, we want to make it easily usable in complex simulation environments where humans interact with robots.

In this work, we have focused on manual material handling (MMH) in warehouse scenarios where human operators handle a defined set of items, and the inertial properties of these items are known. We propose a novel fully-wearable system for biomechanical analysis, which opens up the possibility of analyzing human biomechanics in ecological settings, with minimal impact of the sensing equipment on the performance.

Our algorithm builds on the Recursive Newton-Euler Algorithm (RNEA), recognized as highly computationally efficient due to its low algorithmic complexity [27]. However, we propose a novel tree management that allows loops in the kinematic chain and multiple contact points by differentiating the trees used for the forward kinematics and the backward calculation of wrenches. Differently from conventional implementations of the RNEA (in particular the one implemented in the Orocos Kinematics Dynamics Library), the proposed method divides forward and backward recursion over the kinematic tree. Through this alteration in the inverse dynamics recursion, the system acquires the possibility to manage multiple contact points. A couple of admissible and coherent assumptions have to be considered: the external wrenches on the hands are due to the carried objects, and the contact wrenches on the contact points are known, except for the case of the feet on the ground.

Under these assumptions, this method requires an estimation of external loads. Common approaches require expensive pressure and force sensors applied to smart gloves (e.g., [28]) or insole pressure/force sensors to estimate the ground reaction force (e.g., [29]). Alternatively, EMG and IMU have been adopted [30] for load estimation and they can be used to have an estimation of some articular joint torques, or the carried load [31].

To minimize the cost, the encumbrance, and the discomfort of the sensing devices we propose a novel approach suitable to MMH tasks in which the carried load belongs to a known dictionary. This occurs often in logistics, considering every object contained in a box, its weight is available in a database, and the worker has to scan it whenever it is moved from one location to another.

We propose using an egocentric camera worn on the chest and a computer vision algorithm based on deep learning to recognize the carried box, its identification barcode, and the hand(s) holding it. By moving the complexity from the hardware system to the software and exploiting the object-detection capabilities of state-of-the-art deep-learning-based detectors, we provide a cost-effective though reliable solution for external load estimation.

For estimating the external wrenches due to object manipulation, we exploit the detection of the box and hands, along with a predefined dictionary that allows us to determine the mass of the carried object (identified through the aforementioned barcode). Using the monitored hands’ kinematics, we can also calculate the wrench(es) applied to the hands.

We used ROS to facilitate the integration of our algorithms into more complex simulation setups, even beyond the biomechanical analysis of MMH tasks, for future research.

In summary, the main contributions and novelties of this paper are:

- A novel RNEA-based algorithm that allows for multiple contact points, since it separates forward and backward recursions, performing them with two distinct trees.
- A fully wearable system that collects human kinematic data and recognizes through neural networks the handled objects—from a known database—along with the grasping hand(s) allowing for online biomechanical analysis of MMH tasks.
- The integration of inverse dynamics approach and object/grasp recognition, leading to the ROS implementation—that guarantees remarkably low computing times—of the proposed method that is easily usable with a commercial-off-the-shelf device for motion tracking based on inertial measurement units and a USB camera.

The proposed algorithm has been tested experimentally. The proposed procedure aims to replicate the working conditions of an operator handling and moving boxes in a warehouse-like environment.

The paper is structured as follows: Section 2 shows the algorithm devised for the biomechanical analysis. Section 3 shows the algorithm used for the estimation of the external loads. Section 4 presents the integration of the aforementioned algorithms, whereas Section 5 proposes a preliminary experimental assessment of the method through the analysis of a task that has been performed five times by each of the four selected participants. The paper is concluded by Section 6, which reports a discussion about the overall value of the proposed approach, and Section 7, which summarizes the conclusive assessments.

## 2. Human Biomechanics Model

The model used to represent the human body is inspired by robotics and is composed of rigid bodies connected via revolute joints in a tree-like structure. Several trees are employed to account for different contact points with the external environment, as explained in this section. Each tree is connected to a reference world link by a 6 Degrees of Freedom (DoFs) joint, which is supposed to be fixed. The mass properties of the links are gathered from anthropometric tables [20].

### 2.1. Kinematics and Dynamics Equations

For the implementation of the inverse dynamics, we adopted the Recursive Newton-Euler Algorithm (RNEA), which allows us to easily compute also the articular wrench components which do not make work, and that is flexible for changes of the tree structure. A typical RNEA is composed of a forward and a backward recursion. In the forward recursion, which stems from the root and proceeds towards the leaves of the tree, the link position, velocities and accelerations are computed from the joint kinematics. In the backward recursion, the wrenches exchanged between the links are computed starting from the leaves and going back to the root of the tree. This allows for adding external wrenches at any link and at any time step. Both links kinematic and dynamic equations are written using the spatial Vector algebra, which has been proven to be the most computationally efficient [32]. We use the same notation described in [32]. The velocity and acceleration of the *i*-th link, are $v_{i}$ and $a_{i}$ respectively.

**Figure 1:**
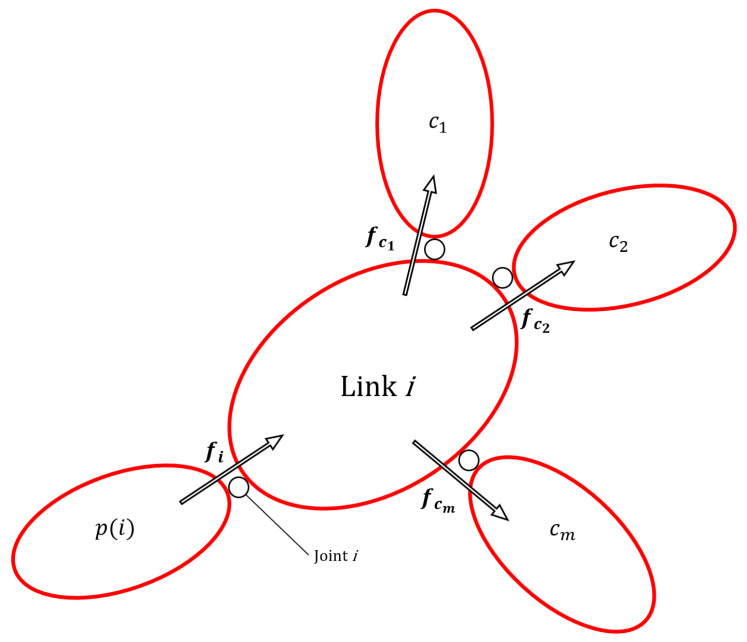
Representation of spatial forces acting on Link *i*.

The matrix $X_{i,p(i)}$ represents the coordinate transform matrix from the reference frame attached to the *i*-th link parent, i.e., *p(i)* and that of the *i*-th link (see Figure 1). $S_{i}$, $q_{i}$, ${\dot{q}}_{i}$, and ${\ddot{q}}_{i}$ are respectively the axis, the position, the velocity, and the acceleration of the joint that connects the parent link *p(i)* to the *i*-th link. $I_{i}$ is the inertia matrix of the *i*-th link. In the forward recursion, the equations of each link kinematics are computed:

(1) $$v_{i}=X_{i,p(i)}v_{p(i)}+S_{i}{\dot{q}}_{i}\;,\;v_{0}=0$$

(2) $$a_{i}=X_{i,p(i)}v_{p(i)}\wedge S_{i}{\dot{q}}_{i}+X_{i,p(i)}a_{p(i)}+S_{i}{\ddot{q}}_{i}\;,\;a_{0}=-g$$

along with the inertial term:

(3) $$f_{i}^{in}=I_{i}a_{i}+v_{i}\wedge I_{i}v_{i}$$

which is used in the backward recursion, in the i-th link dynamics equation.

In the backward recursion, first the force balance equation of each link are computed, thus obtaining the wrench applied by link *p(i)* to link *i* via joint *i*:

(4) $$f_{i}=f_{i}^{in}-f_{i}^{e}+\sum_{k\in c(i)}X_{ik}^{*}f_{k}$$

where $f_{i}^{e}$ is the external wrench applied to the *i*-th link, $f_{k}$ are the wrenches applied by the *i*-th link to its children c(k) and $X_{ik}^{*}$ is the transformation that allows moving a wrench written in the frame *k* to the *i*-th link reference frame. Finally, the torque applied by joint *i* is:

(5) $$\tau_{i}=S_{i}^{T}f_{i}$$

### 2.2. Wrenches

The wrenches that the human body exchanges with the external world and those due to internal loops are separated into two classes: those due to the object carried by the user and those due to contact with the environment. The former is estimated by employing deep learning techniques, as shown in Section 3. In the cases in which only one contact point occurs, there is no redundancy and the wrench at the contact point is obtained by attaching the world link at the contact point. If more contact points occur, the related wrenches cannot be uniquely determined by inverse dynamics.

In this paper, we assume that these wrenches, except in the case of double support in gait, are known. These wrenches can be obtained using sensors (e.g., pressure sensors in smart textiles), using heuristics, or by optimization procedure, but this is not the focus of this paper since in object manipulation it is unlikely to have multiple contact points different from the contact of the feet with the ground.

### 2.3. Tree Management

**Figure 2:**
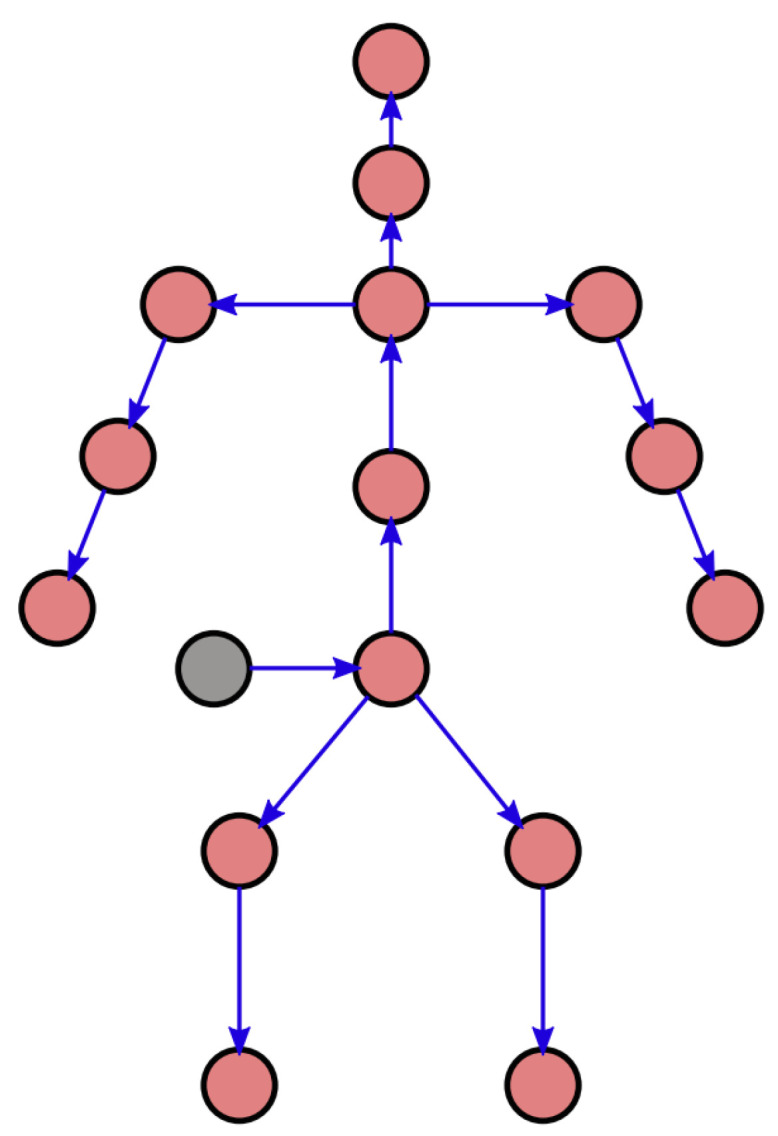
Computational flow during outward recursion. The red circles represent rigid bodies, whereas the blue arrows represent the joints connecting them. The grey circle represents the world and functions as a starting condition for the outward recursion.

Differently from the state-of-the-art RNEA implementation used in the Orocos Kinematics and Dynamics Library (KDL), we propose to separate the forward and the backward recursions in the algorithm and perform them with two distinct trees. This allows us to cope with multiple contact points and to perform the RNEA with any tree structure. Since the external wrenches are calculated with respect to the world link, whereas most of the motion capture systems are rooted in the pelvis, we use different trees for the forward and the backward recursions according to the contact point. Although the mass properties of the link of these trees are always the same, the link hierarchy has to be changed according to which link is connected to the world link. The tree used for the forward recursion is shown in Figure 2. The world link is attached to the pelvis because most of the motion capture systems are rooted in the pelvis.

A single joint with six joint variables is displayed to represent the fictitious six joints corresponding to the pelvis’ degrees of freedom with respect to the world link. Each of the green joints is associated with three revolute joints in a wrist configuration. This solution is consistent with the BVH (Biovision Hierarchy Animation File) motion representation standard, which is commonly available in motion capture systems. For the backward recursion, several trees are possible.

In this paper, we focus on the special cases of MMH in which the worker walks or stands on two legs while carrying and manipulating a cardboard box. The tree used for the backward recursion changes according to the current contact points with the environment. In particular, its root link coincides with the human body link that is in contact with the environment.

**Figure 3:**
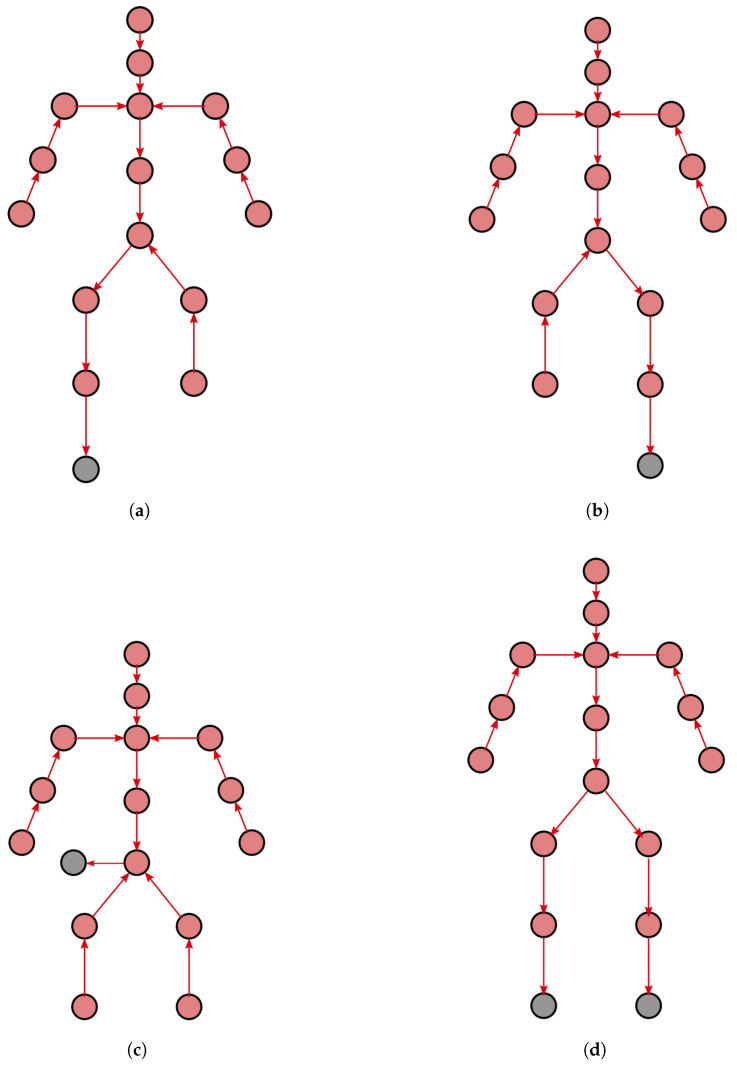
Trees used in the four gait phases. The grey circles represent the world link. Arrows represent the computational flow from the leaves to the root. (**a**) Left foot support; (**b**) Right foot support; (**c**) No support; (**d**) Double support.

The selection of which tree should be used for the backward recursion is based on the feet’ kinematics. Gait includes four possible phases: one-leg support, double-leg support, and no-leg support. Therefore, four trees are used, one for each contact configuration. The backward trees used in the backward recursion are reported in Figure 3. Currently, the logic used for the gait segmentation simply applies a threshold on the ankle vertical distance from the ground. We plan to use more algorithms to determine foot contacts with the ground at each instant, but this is not the focus of this paper.

The indeterminacy that occurs at the pelvis when both feet are in touch with the ground is dealt with as follows. First, the force computation proceeds from the upper limbs towards the pelvis. At the hips level, the wrench is distributed on each leg based on the body’s center of gravity position to ensure static balance as if the legs had no mass. Finally, each leg is solved separately.

A better estimation of the contact force with the ground may be obtained through pressure sensors, but the proposed solution is much simpler from a hardware point of view and it is reasonable for low-speed walking and for operations in which the worker manipulates the object in a quasi-static configuration.

### 2.4. Implementation Issues

The implementation of this method moves from the motion data, that we suppose to have available in BVH format. This format does not include inertial information of the links, but only the kinematic structure. Therefore, we decided to map the BVH representation of the human in a Unified Robot Description Format (URDF) to include the inertial properties in a format that is used in ROS, thus easing the future development of simulations that include robots interacting with humans. This solution allows us to include the hierarchy of the links that will be used in the forward recursion.

Since the forward recursion and the backward recursion for relevant contact points configurations require many trees, which differ only for the position of the world link and for the hierarchy, a function that generates all the required URDF files from the same BVH file was developed.

The last implementation issue regards the hierarchies in the forward and backward recursions. We observed that the best choice for reference systems definition, in terms of computational efficiency and code clarity, was to keep the tree reference systems of the forward recursion. The Newton-Euler equations of each link are always written in the reference systems of the forward tree. The output of each backward recursion step is the spatial force that the parent link (in the backward tree) exerts on the child. The backward recursion is modified to ensure that the equation of motion of each link is expressed in such reference systems and to guarantee the correct force computational flow.

## 3. Load Estimation

In MMH activities it is reasonable to assume that most of the external load is due to an object carried by the hands of the user. To estimate such load, we propose a method based on an egocentric camera and deep learning algorithms for object and grasp recognition. An egocentric camera is placed on the chest of the user so that the hands and the object are in the field of view during object manipulation.

The frames obtained with the camera are processed by the object detection model YOLO [33], a state-of-the-art deep learning neural network. In particular, in this project, the model *nano* [34] of YOLOv8 [35] (at the time of writing it is the latest version of the YOLO object detection model) is trained and deployed.

The proposed method is applied in a situation that aims at reproducing the conditions of an operator that moves packages in a warehouse. Different cardboard boxes were selected; each of them has been equipped with a barcode that identifies the product. The identified object is an item in a database that includes data about the object’s size and mass (a common situation in logistics). To detect and track the boxes during the experiment, the *box-6500images* (https://universe.roboflow.com/project-9tdw1/box-6500images, accessed on 30 March 2023) dataset has been used for training the selected object detection model. The cardboards dataset has been split into a training set (80% or 3188 images), a validation set (10% or 390 images), and a testing set (10% or 391 images). The model achieves an F1-score of 0.91.

For detecting the hands of the human operator another nano YOLOv8 model pre-trained on the popular dataset COCO [36] has been used. Regarding the evaluation model used for person detection, with the help of Fiftyone (https://github.com/voxel51/fiftyone, accessed on 15 April 2023) library is possible to inspect the performance metrics of the given model. The F1-score for the category person is 0.76.

For detecting and decoding the barcode OpenCV library (https://docs.opencv.org/4.x/d6/d25/tutorial_barcode_detect_and_decode.html, accessed on 20 March 2023) has been used, creating an instance of *cv2.barcode.BarcodeDetector* and executing the function *detectAndDecode*.

The biggest problem encountered during the test of a single network, after the training on the two separate datasets, is the absence of labels regarding the other category. Different experimental results have reported greater accuracy and smaller inference times using two mono-class models with fewer parameters instead of a single more complex version trained on the merged datasets. For this reason, two nano YOLOv8 models have been used in parallel: one for detecting people (hands result as a part of a ‘person’ object) and the other for identifying cardboard boxes.

Then, we performed an additional analysis of the bounding box center with respect to the frame. Given the camera position, it is reasonable to assume that the left and right hands will most likely be on their respective side of the image. The bounding boxes that enclose the hands and the object detected in the image are analyzed. If the Intersection over Union (IoU) of a hand box and the object box is higher than a threshold value, the object is recognized as grasped by that hand.

We also implemented a state machine in which the object grasp conditions are coded. One object can be either handed by no hand, left hand, right hand, or both hands. The object enters one state according to the aforementioned IoU score computed for each hand. The transition to another state is only allowed when a time threshold of stay-in-the-state is exceeded.

**Figure 4:**
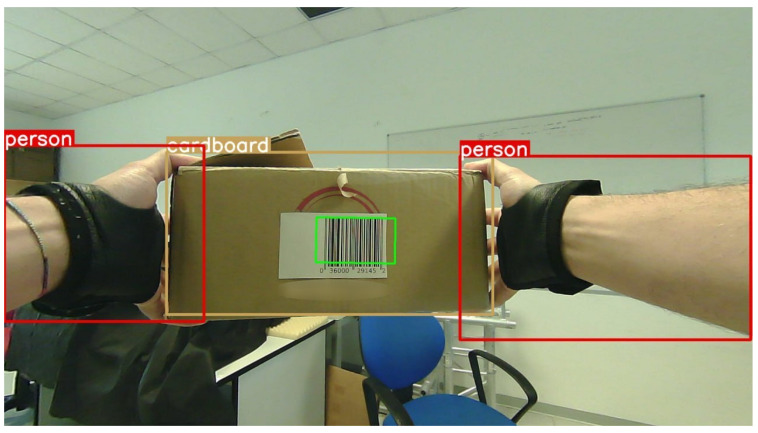
Example of a frame, from one of the videos recorded during the experiments, reporting the output of the detection of the operator’s hands, the carried box, and the identifying barcode.

In Figure 4 the bounding boxes are reported on a frame (taken from a video recorded during one of the conducted experiments) where a card-board box is grasped with both hands; also the detected barcode for the identification is highlighted.

We created a dictionary containing, for each of the barcodes attached to the boxes, the respective inertial information necessary to compute the external wrench on the hands.

This includes:

- the type of object that is carried and its inertial properties, i.e., mass and barycentric inertia tensor
- the coordinate transformation from the hand reference frame to the barycentric inertia system. This information can vary, so a list of transformations in typical grasp conditions for each object was created (one transformation matrix $X_{hg}$ for each condition)

In the case of both hands holding the object, we shared the load equally on the hands. The load that the hand exerts on the object can be computed as:

(6) $$f_{H\overset{}{\to }G}=X_{hg}^{*}I_{G}X_{gh}a_{H}+v_{H}\wedge \left(X_{hg}^{*}I_{G}X_{gh}v_{H}\right)$$

where $f_{H\overset{}{\to }G}$ is the wrench that the hand exerts on the object, $X_{hg}$ is the transformation from the object barycentric inertia tensor to the hand frame, $I_{G}$ is the object inertia tensor, $a_{H}$ and $v_{H}$ are the hand acceleration and velocity. This is true in the reasonable hypothesis that the object does not move relative to the hand that holds it, which is true for most rigid carried objects.

## 4. Integration in ROS

**Figure 5:**
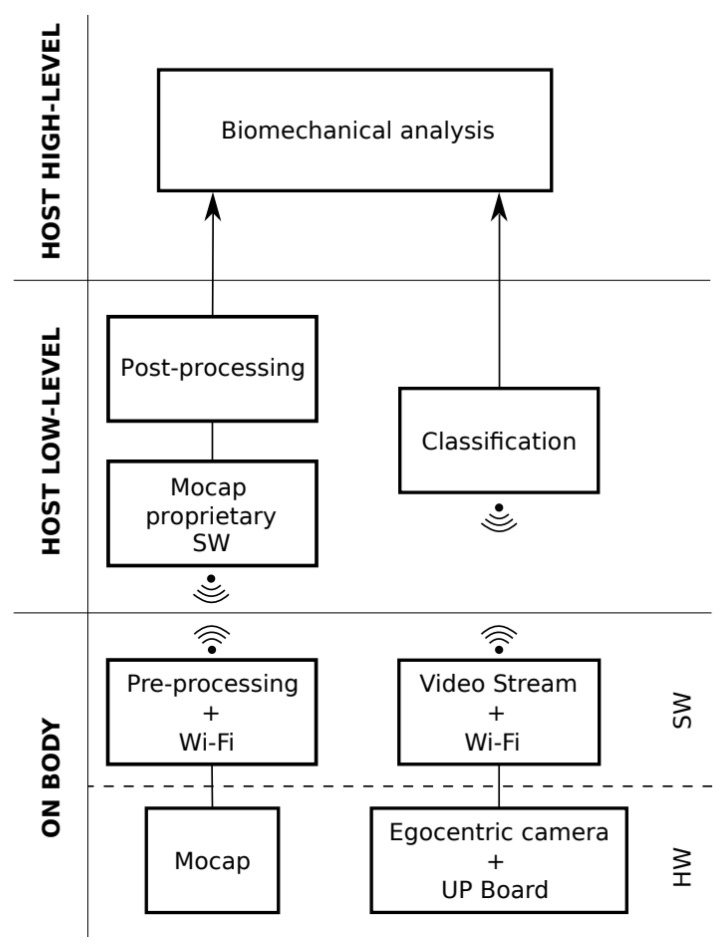
Biomechanical analysis system architecture.

The inverse dynamics algorithm described in Section 2 and the load estimation pipeline described in Section 3 were joined into a single system that performs the biomechanical analysis. The system architecture is shown in Figure 5.

It is composed of two parts. The one on the bottom includes the devices which are worn by the user and the processing steps which are executed by these devices. The upper parts show the software modules, which run on a host PC, to execute the biomechanical analysis. The same architecture reports two data processing pipelines.

The left one provides the kinematic data. At the bottom, the Mocap module represents the motion-tracking system that is worn by the user. In the current implementation, either the Xsens MVN -Link-Biomech tracking system (Enschede, 7521, The Netherlands) or the Noitom Perception Neuron (Miami, FL, 33137, USA) Both are composed of 9-axes Inertial Measurement Units equipped with magnetometers. The raw data coming from the are Pre-processed by a proprietary software module (either the MVN Analyze if the Xsens is used or the Axis Neuron v3.5.24 if the Perception neuron is used) and sent via Wi-Fi to a local host. In this pipeline, the local host runs proprietary software which processes motion data and returns motion data in BVH format. This output is then post-processed to provide joint angles, velocities, and accelerations to the biomechanical analysis module. Before differentiating the joint angle variables through the backward Euler method for obtaining the velocities and accelerations, a 12th-order LowPass Butterworth Filter is used with a cut-off frequency of 80 Hz.

The right pipeline corresponds to the vision data. On the user body, the egocentric camera (ELP-USBFHD06HBL180 fish-eye camera) is connected via USB to a UP Board (UP Board RE-UP-CHT01-A10-0116) which features Ubuntu 18.04 bionic and OpenCV version 3.0. The camera sends H264 compressed video to the embedded board that records video and sends the acquired data to the local host via Wi-Fi. This Wi-Fi separation makes the acquisition system completely wearable. The streamed video is the input of the Object classification module, which runs on the host PC. This module processes the video using the mentioned neural networks to obtain the grasp information, i.e., the object being held and which hands hold it. The object and the pick information are used to select the right information from the barcode dictionary and send them to the biomechanical analysis module. This latter processes motion data and the information about the carried object to select the proper tree and compute the wrenches at every link and the torques at every joint, which is the final output of the system.

**Figure 6:**
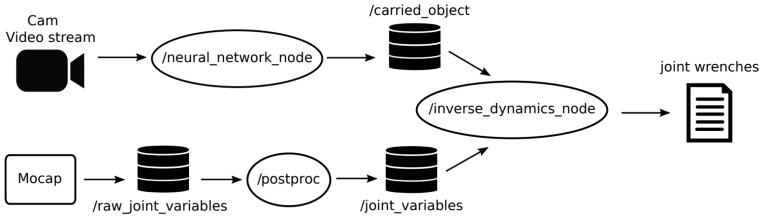
ROS scheme in online mode.

Figure 6 shows the ROS implementation of our system. The neural network node is a ROS node written in Python language. It runs the CNNs on the frames that come from the camera video stream. The combined neural networks take roughly 15 ms to process each video frame. This node also implements the additional functions that analyze the bounding boxes obtained with the neural networks. The output of this node is published on a ROS topic that stores the grasp corresponding to each frame.

The joint variables obtained with the motion tracking system (i.e., the output of the proprietary mocap software) are processed by the post-processing node and given as input to the biomechanical analysis node, which is written in C++.

The whole system can run either in online or offline mode. In the latter case, the topics, the video stream, and the motion data stream are replaced with files that store the gathered data.

## 5. Experimental Assessment

### 5.1. Experimental Procedure

**Figure 7:**
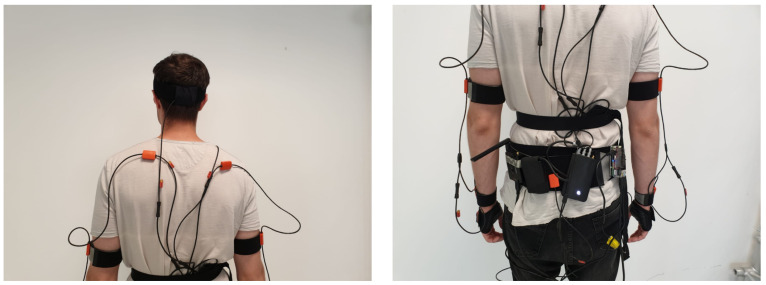
Experimental Setup.

The whole system was tested during a study case performed in laboratory settings that aims at replicating the warehouse environment. Four healthy volunteers participated in this experiment. Participants did not report issues related to the musculoskeletal system or that could prevent completing the task, they were aged 31 ± 8, tall 1.70 ± 0.08 m, and with mass 69 ± 13 Kg. They were asked to wear the Xsens MVN-Biomech suite as shown in Figure 7 along with the egocentric camera, the UP Board, and a power bank. These latter were fixed to the chest using an elastic band.

The participants were instructed about the task and the goal of the experiment, and age, anthropometric measures, and weight were annotated.

**Figure 8:**
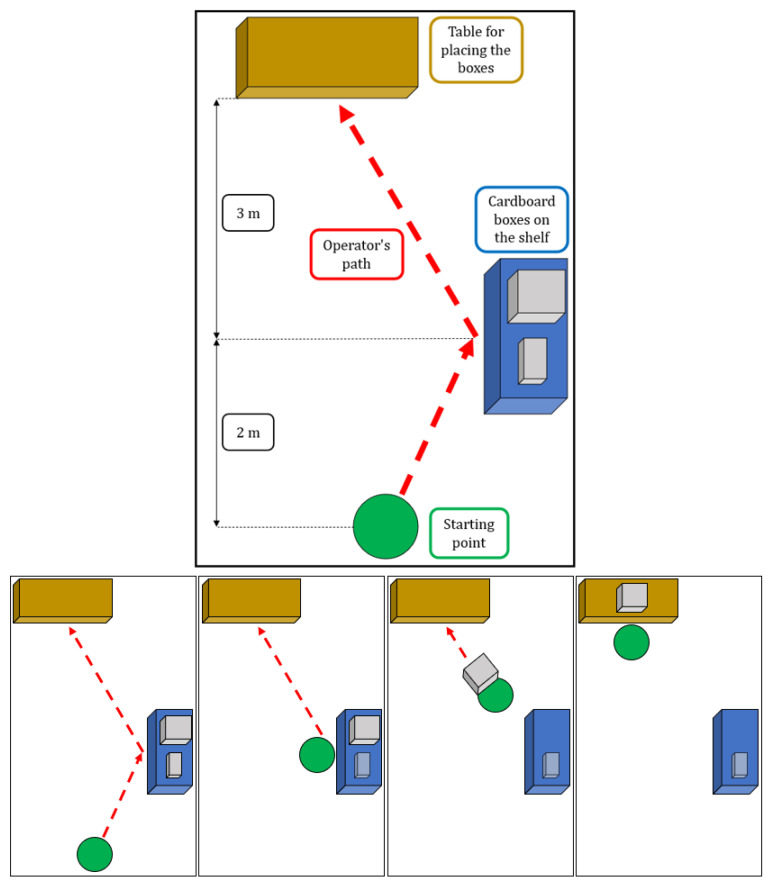
Simple graphic representation of the environment in which the experiments were conducted, with a basic delineation of the path walked by the participants and the phases of the task.

After a brief calibration procedure needed to set up the motion capture system, they were asked to reach a starting position marked on the ground and stand still in N-pose (stand with arms alongside the trunk). Then they had to walk for 2 m to reach a desk where they had to pick a box with both hands. After picking the box, they had to bring it to another table 3 m away from the first one and release the box. This experimental procedure is represented in Figure 8.

The host pc used for the experiment is an MSI GF63 Thin 10SCSR-073IT Notebook ( Intel Core I7-10750H, 16GB RAM, GPU Nvidia GTX 1650Ti). Figure 7 shows the experimental setup, with the participant wearing the XSENS sensory system.

This cycle was repeated five times, and each cycle lasted about 15 s on average. The duration of each experiment varies depending on the self-selected speed at which the participants performed each task (for example, some take more time for the grabbing phase, while others walk lightly slower when carrying the box).

### 5.2. Data Processing

Anthropometric data and the mass of each participant were used in the initialization phase of this algorithm to generate the URDF file that represents the digital twin of the participant.

Motion data were processed online by the XSens MVN Animate software, which ran on the host PC, to locally stream joint angles. These angles were then used to produce the joint speeds and accelerations according to the method reported in Section 4. At the same time, Camera images were streamed to the host PC that ran the detectors and provided the external wrenches. We estimated 80 ± 20 ms latency between motion data and wrenches. Therefore we temporally aligned motion data to the wrenches obtained from the camera images analysis.

Finally, we stored the results of the detectors and of the biomechanical analysis to evaluate the system performance. In the following section, we report plots that show some of the forces and torques estimated during the conducted experiments. Besides the examination of data related to a single experimental procedure, a comparison is performed between the mean values of the examined forces and torques computed for each of the participants along the five repetitions of the task described previously. As stated before, each experiment has been conducted without imposing time constraints, therefore a certain variation can be observed even between repetitions executed by the same participant. To compare the variables related to different task executions and various participants and calculate the mean of the variables of interest, a temporal synchronization has been performed for the offline processing of the data, exploiting the *alingnsignals* functions from MATLAB to align the signals. The computed variables are originally composed of a dissimilar number of samples given that different executions of the same task require distinct time intervals. Therefore, before performing the alignment, these time functions have been normalized with respect to the task accomplishment. Then the mean values have been calculated and reported in the following section, allowing the comparison between experiments of multiple time durations and performed by different participants.

### 5.3. Results

The captured data were used to test the load estimation algorithm. We report in this section the estimation of the wrenches at relevant joints, focusing first on the information related to a single experimental procedure, and concluding with a comparison between data of different participants. The variables reported in the following are expressed in the local frames of the investigated joints, arranged according to the *bvh* standard, in which the *y* axis is directed like the main limbic axis.

**Figure 9:**
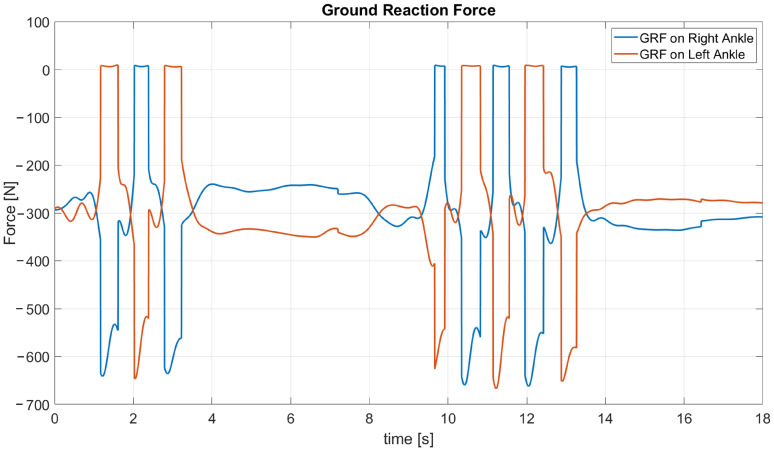
Vertical force computed online at the right ankle joint, from one of the task executions of participant 1.

As stated before, the current gait segmentation approach is based on the height of the ankle joint, using a threshold that has proven itself quite accurate in the detection of the steps during the task, even if a better gait segmentation algorithm is necessary, and will be the next focus of this work. Concerning the ankle joint, in Figure 9 the forces exerted on the right and left ankles by the ground are reported.

As can be stated from the overlapped variables, the steps performed by the participant are detected, and the value of the peaks of the ground reaction forces are coherent with the masses of the user and the carried box. Given the sufficient recognition of the steps during the experiments, we can identify the first three steps (without load) that the participant performs for reaching the shelf with the boxes. Once the box has been grasped, five other steps are necessary for getting to the table where the object will be left. In the second group of steps, a slightly bigger magnitude of the peaks can be noted, given that the participants that is carrying the 2 Kg box has a mass of 64 Kg.

During the first seconds the participant stand in the N pose, as stated in the protocol. From seconds 1 to 3 he walks to the shelf where the cardboard boxes are. He stands in front of the shelf for about 6 s, and after this period, he has grabbed and lifted the selected box and then starts walking to the table where he will leave the box. The details regarding the grabbing and lifting movements are going to be clear by analyzing the forces and torques at the upper limbs. From second 9 to 13 he walks to the table (the distance is higher, so it takes five steps to walk this part of the path). As for the previous stand phase, the details about the actions from the second 13 to 18 will be investigated through upper limb dynamics.

This preliminary recognition of the phases of the experimental procedure is useful for examining in an effective way the other results of the inverse dynamics algorithm, such as slight oscillations in the forces and torques at certain joints due to the walking movements.

**Figure 10:**
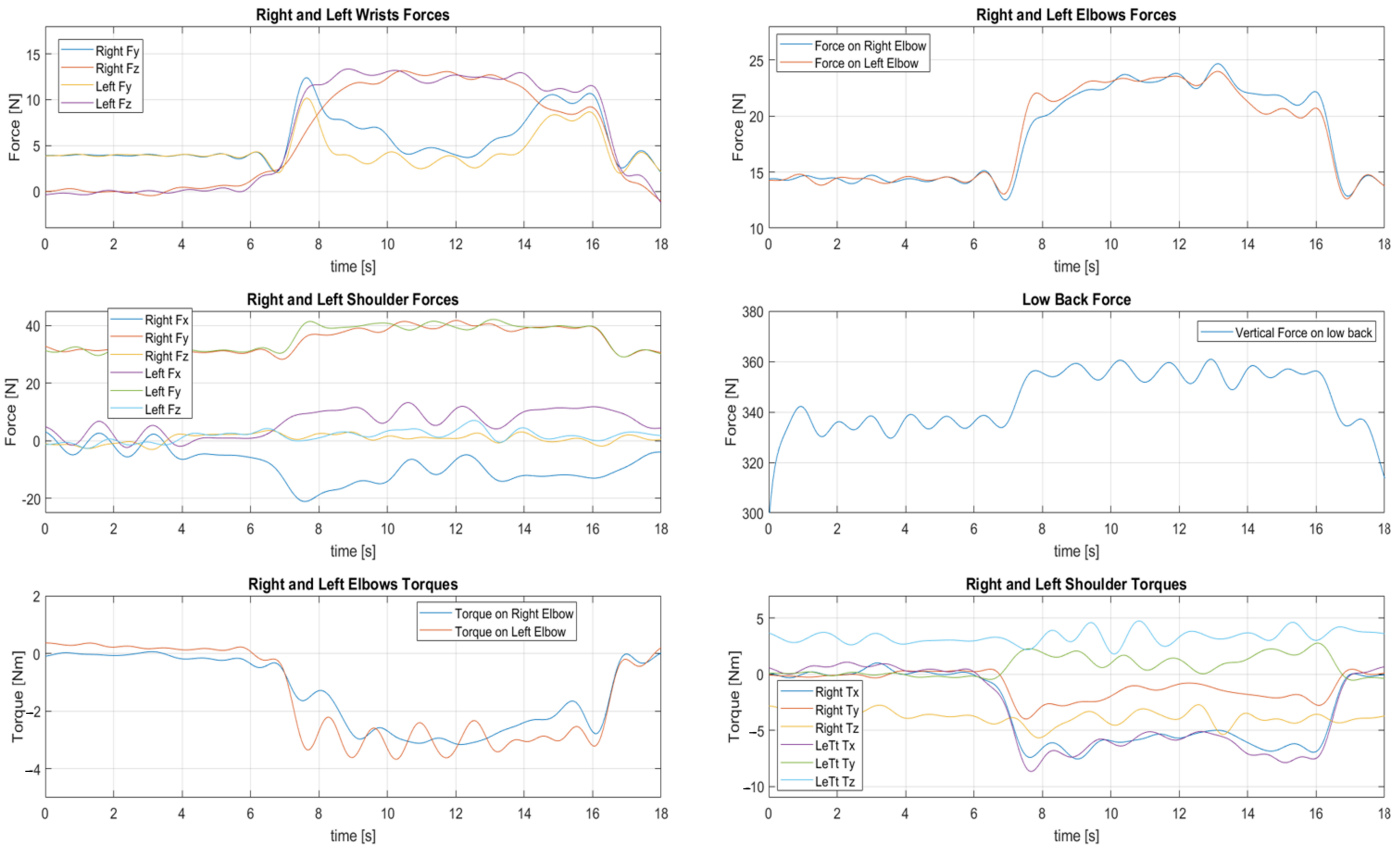
Selection of some of the computed forces and torques for one of the task executions performed by the participant 1.

Regarding the upper human joints, the following panel of graphs in Figure 10 represents some of the forces and torques computed at the wrists (the pictures in position (1, 1) in the panel), elbows ((1, 2) and (3, 1) in the panel), shoulders ((2, 1) and (3, 2) in the panel), and lower back ((2, 2) in the panel) of participant 1, that is 1.70 m tall, aged 28, and has a mass of 64 Kg. Some forces or torques are reported only along some directions (as in the pictures of the panel regarding the elbows) for showing only the most interesting variables.

Regarding the Wrist forces, before the grasping movement gravity has the main contribution (the forces are coherent with the mass of the hand in the model, that is 0.4 Kg). While holding the box, the wrists rotate, moving the effect of gravity on the *z* component. The phases where the forces in both *y* and *z* directions are about 8 to 13 N coincide with the grabbing and dropping activities, so when the participant brings the box near himself from the shelf and turns it away on the target table. In these instants, the accelerations are not only due to gravity but to the participant’s motion.

A consistent correlation can be observed between the forces at the wrists and those at the elbows and shoulders. The synchronous plateaus from seconds 8 to 16 give us information about the exact grabbing and dropping actions, with correct differences in the modules because the upstream joints have higher masses attached. A similar assessment can be proposed regarding the lower back vertical force, whose increment is coherent with the before-mentioned plateaus, and the extent of the increase confirms the presence of 2 Kg mass of the carried box.

Considering also the estimated shoulders’ torques, it can be observed how the grabbing (at the beginning) and dropping (at the end) phases can be distinguished from the load-carrying phase, noting the peaks—due to the arm extensions in the mentioned phases—of the plateaus of the variables along the *x* direction.

For concluding this preliminary analysis of the results related to a single participant, it is important to note, in the charts reporting variables from right and left body parts, the similarity between these variables, be they analogous in modulus and sign (e.g., shoulders’ forces along *y* and the forces on elbows) or just in module (e.g., the shoulders’ torques along *y* and *z*).

**Figure 11:**
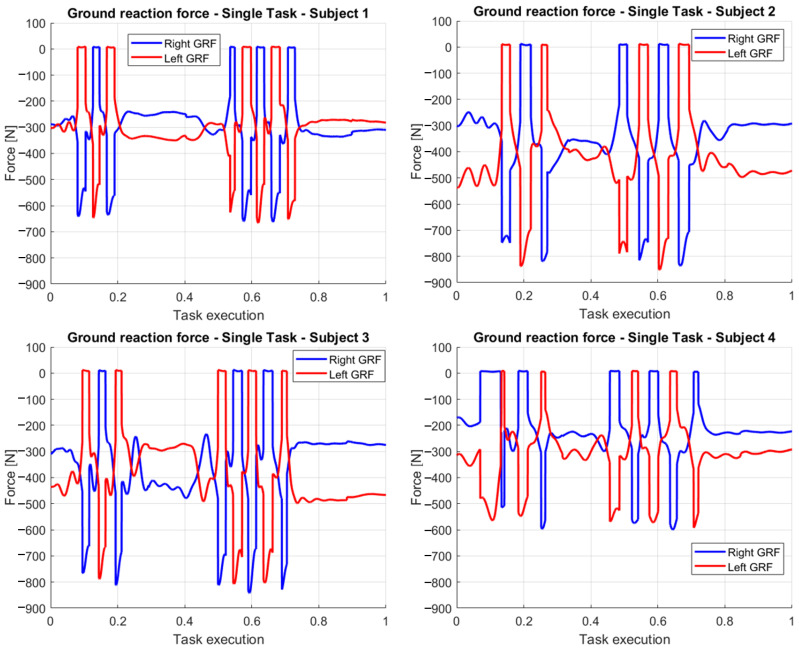
Ground reaction forces at the right and left ankles of the four participants, reported normalized with respect to the task execution.

After the presentation of the results regarding participant 1 during a single experiment, in the following figures, we report a comparison between forces and torques computed in case of different participants. In Figure 11 the ground reaction forces are estimated at the right and left ankle in one experimental task for each participant. Through these pictures, we can observe how the different masses (e.g., participant 1 weights 62 Kg and participant 2 weights 82 Kg) of the participant lead to consistent ground reaction forces, showing also a little increase after the grasp due to the weight of the carried box. The number of steps estimated through the inverse dynamics algorithm is consistent in every analyzed experiment with the procedures performed (the tallest participant executes fewer and longer steps). For the first three participants, the gait segmentation approach based on an ankle height threshold works quite accurately, while it shows some flaws with participant 4, who is 1.62 m tall, the smallest height of the four participants.).

The graphs regarding the ground reaction forces are also useful for identifying the phases of the task, helping in the interpretation of the other variables. We can observe, for example, that the participants take a different amount of time for each of the transitions that compose the task.

**Figure 12:**
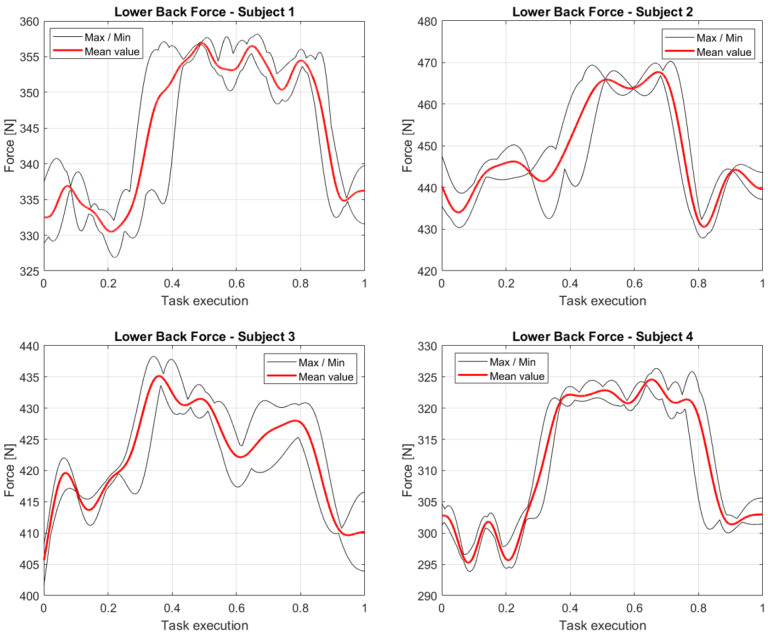
Lower back vertical forces, normalized with respect to the task execution, computed as the mean of the variables collected during the five repetitions of the assignment by the four participants.

In Figure 12 the main components of lower back vertical forces are reported, after being processed as explained in the previous section. As expected, the forces are consistent with the masses of the participants and the box. The mean red lines are computed after the normalization (with respect to the task execution) and the alignment of all the data available for a precise participant. The final falling edge represents the instant when the participant drops the box on the target table diminishing the vertical load. The alignment works more effectively on that because the dropping of the boxes was the triggering event for stopping the recording. On the contrary, given that different velocities of execution have been observed in all the performances of the four participants, the mean rising edge has a major margin between the maximum and the minimum limit. For example, participant 2 shows the narrower plateaus, meaning that he was the fastest at taking the box, carrying it to the table, and dropping it. On the contrary, participant 4 and 1 shows larger plateaus, with differences in the uncertainties on the rising edge, meaning that participant 4 performed the task approximately at the same rate all five times. The force profile that differs the most from the others is the one relative to participant 3. Evaluating the other two forces at the examined joint, we noted how the mentioned participant would tend to slightly lean forward, diminishing the force in the investigated direction.

**Figure 13:**
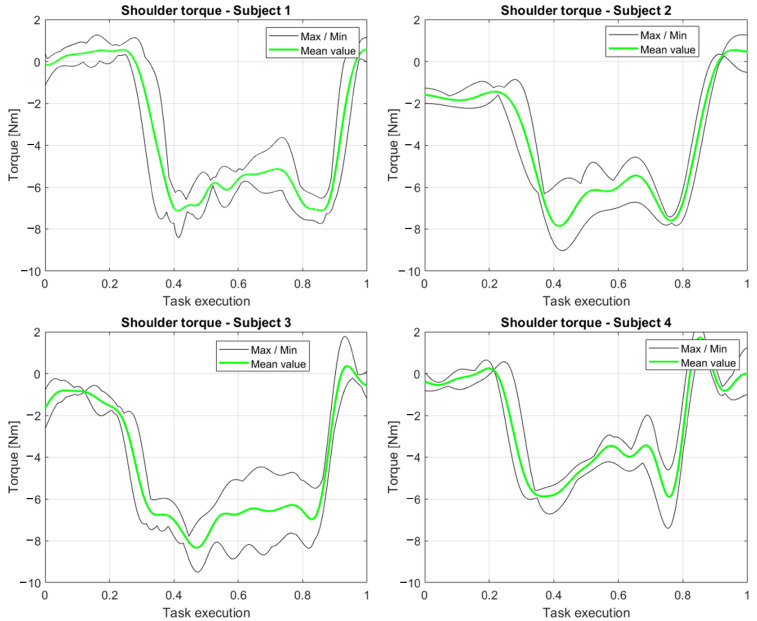
Shoulder torques, normalized with respect to the task execution, computed as the mean of the variables collected during the five repetitions of the assignment by the four participants.

Figure 13 reports the estimated torques at the shoulder joints of the four participants. In these plots, the starting and ending peaks that can be recognized at the limit of the plateaus represent the instants of elongation of the arms for taking and dropping the box. The final positive peaks (observed in participants 3 and 4) show the fastest retreat of the arms once the box was left on the table, consistent with how the experiments have been conducted by the participants. Even if limited in the module, the differences between the torques’ values are motivated by the dissimilarity of the anthropometric measures between the participants. For example, the fourth and shortest participant (therefore with the shortest arms for the torques) shows minor torques with respect to the tallest (participant 2, 1.78m tall). This difference is also due to the discrepancy in the movements performed by the participants.

**Figure 14:**
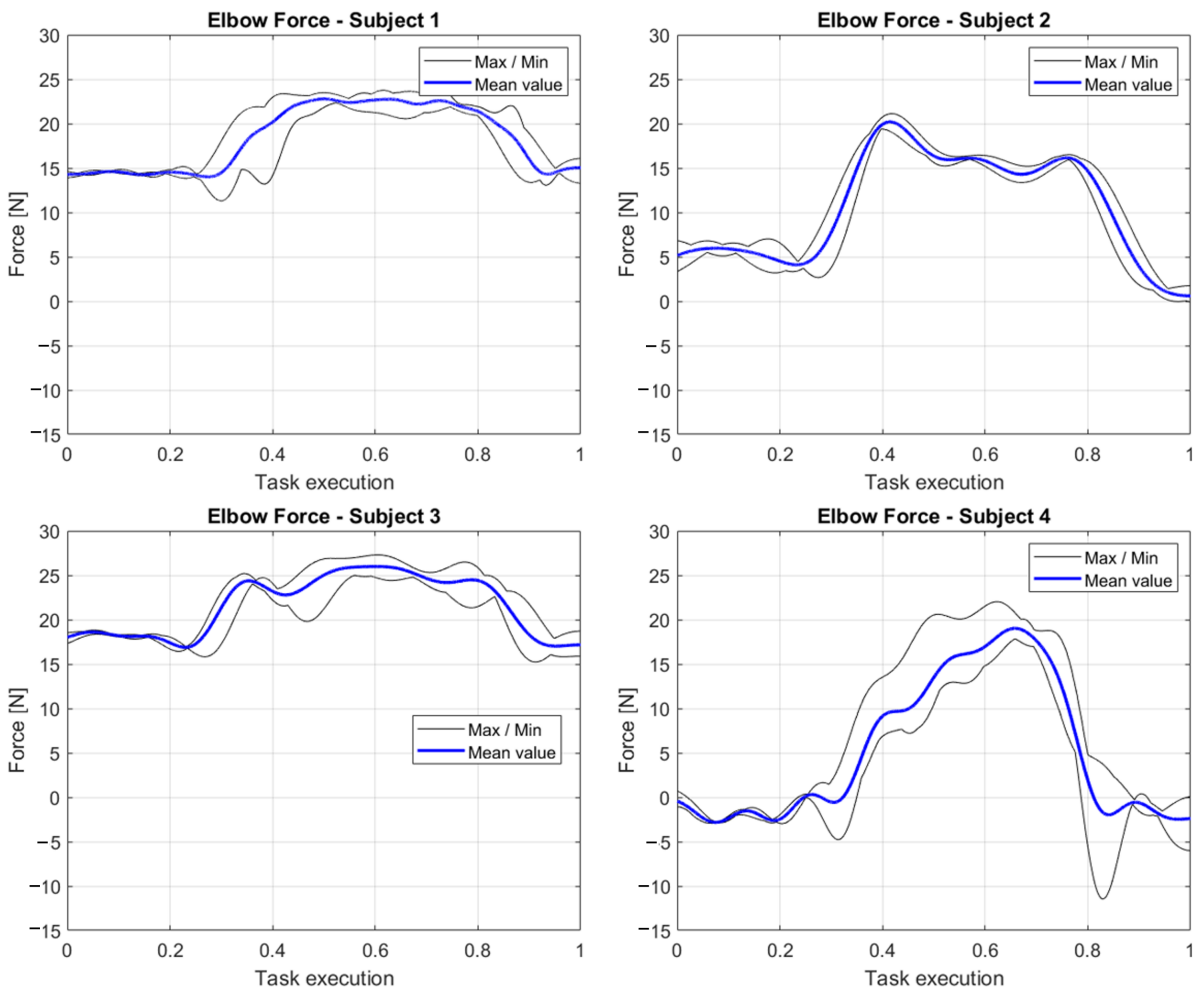
Elbow forces, normalized with respect to the task execution, computed as the mean of the variables collected during the five repetitions of the assignment by the four participants.

In Figure 14 we report the components of elbows forces which better express the variations through the task due to the grasping and carrying of the object. Indeed, the graphs reporting the data of participants 1 and 3 are quite similar, starting with a force that represents the weight of the forearm. Concerning the elbows’ forces reported for participants 2 and 4, it can be noted - through the evaluation of the other components of the wrench at these joints - how the difference in the poses and motion joints trajectories for accomplishing the tasks can be extrapolated by interpreting the data from our inverse dynamics approach. The fact that the examined element of the force does not start with a value coherent with the mass of the forearm means that, during the grasping-carrying-dropping sequence, participants 2 and 4 rotate their arms, adjusting a self-selected position for a more comfortable pose to maintain while holding the box. The force profile that differs the most from the others is the one relative to participant 4. Through an examination of the other two forces at the elbows’ joints, we observed how this participant not only rotates the elbows for interacting with the box, but also tends to tilt the elbows in the first stage of the grasping and load-carrying phases, initially diminishing the force in the investigated direction.

**Figure 15:**
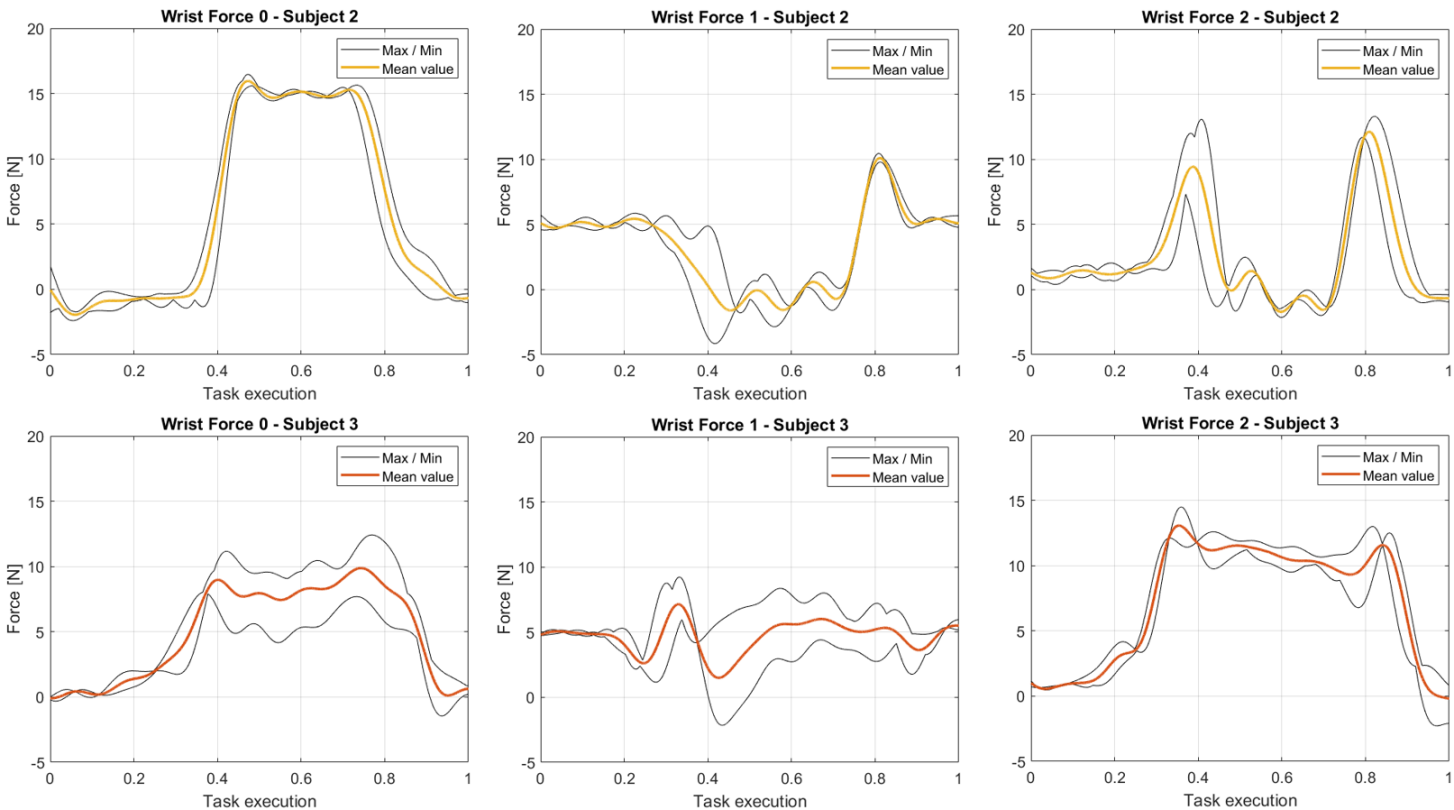
Wrist forces, normalized with respect to the task execution, computed as the mean of the variables—corresponding to the three components of this force—collected during the five repetitions of the assignment by participants 2 and 3.

To present an example that shows how the rotation of the investigated articulation during the task affects the forces estimated, in Figure 15 we report the mean (together with the maximum and minimum limits) of the three components of the force at the wrists for the participants 2 and 3, normalized along the execution of the experimental procedure repeated for five times. The modules of the proposed forces are consistent with the masses of the body segments of interest (in this case the hands) and with the dynamics of the events during the procedure. Element 1 of the Force reported (that is to say the two graphs in the middle column of the panel) contain the main contribution in the unloaded walk (before taking the box), i.e., the gravitational component, however, the different behaviors of the examined participants lead to dissimilarities in the subsequent trends.

As mentioned previously, by analyzing the components of this force, we can investigate distinct variations of the grasping, dropping, and load-carrying hand pose. In this case, participant 2 presents two spikes in Force 2 at the moments of grabbing and depositing the box, with a continuous and higher plateau on the 0 component. This means that the self-selected hand pose varies during the phase of carrying the load from the shelf to the target table. On the contrary, evaluating the elements of the force at the wrist of participant 3, we observe that two plateaus with similar modules coexist from the grasping moment to the final dropping stage. This means that participant 3 maintains approximately the same position and orientation of the hands while interacting with the box.

Observing the correlation between the data related to the joints of the same arm, it can be noted how the backward recursion effectively conveys the forces and torques from the last joint of the human chain (in this work the wrist) to the other joints connected in series with it.

## 6. Discussion

The experiments have proven that the neural networks were properly trained, performing very well in the hands and box recognition. Furthermore, the implementation of a finite state machine permitted to avoid the wrench discontinuities when the camera loses the targets, which occurs when the operator’s hands are not detected, the carried box is not recognized or its identification barcode is not identified in the image.

The choice of the camera is satisfactory, indeed the load estimation performed with the aid of an egocentric camera seems to be a good approach, making it suitable for the case of people that have to work with objects with similar appearance whose content (therefore inertial properties) can be identified through the detection of a certain element (in this case barcodes).

The proposed RNEA algorithm easily allows for the application of external wrenches on any body of the tree thanks to the novel tree management described in Section 2.3. The experimental results are consistent with the gait analysis results that can be found in the literature (e.g., [37]) both in terms of absolute values of ground reaction forces and their distribution over the gait cycle. Results show that the system copes correctly with strongly differently sized participants, providing a consistent estimation of wrenches regardless of the user’s anthropometric measures. Articular forces and torques are also consistent with the literature for similar tasks, even if an immediate comparison is not feasible. Indeed, examining similar works from the literature, such as [38,39,40], both the experimental protocols and the way of displaying the results (not showing forces and torques but EMG data or fatigue factors) turn out to be quite different. In [41], for example, the task of lifting is performed with heavier boxes and following a quite dissimilar movement pattern. We draw a similar conclusion considering the results from [42], where the torques at wrist and elbow joints are reported from quasi-static experiments conducted using two different loads. Consequently, only a quantitative comparison is allowed, while considering a scaling factor that accounts for the difference in the manipulated load and anthropometric measures. In the proposed ROS implementation, the inverse dynamics solver executes within the desired rates with its 2 ms computation time. This perfectly fits with the desired objective of performing an online biomechanical analysis.

The experiments, conducted with a sufficiently variable set of participants, allowed us to stress the current limitations of this approach, which are mainly due to the simple algorithm used for gait segmentation, which is based on a threshold on the ankle position. A more complex gait classification, e.g., a classifier based on the joints angles, could be used to improve the leg wrenches results without using costly and uncomfortable insole sensors and avoid discontinuities. This will be the target of future studies to improve the novel system proposed in this paper. The mentioned limitation does not apply to the upper body part, where the wrenches have proven to be consistent for every upper body joint.

Besides enhancing the gait segmentation system, future insights will lead to investigating the method exposed in this paper through extensive experimental validation for other tasks that are exhaustively analyzed in the literature. Therefore, not only the MMH in a warehouse-like environment for logistics applications will be deepened by enrolling real-world operators, but also other experimental procedures are going to be examined to extend the possible confrontations with other works from the literature.

## 7. Conclusions

The paper presented a novel method for the biomechanical analysis of the human that naturally allows for the application of multiple wrenches, and is particularly suited for the case of manual material handling activities in a warehouse-like environment. The results show that the idea of using an egocentric camera is suitable for online load estimation, which needs efficient and computationally light neural networks for the required detections, in high-impact cases such as those in logistics. The whole system can perform a correct biomechanical analysis of the user’s activity, though improvement is necessary to have a more reliable gait segmentation and an experimental exploration of different tasks, aiming at a more comprehensive confrontation with the literature.

## Data Availability

The data presented in this study are available on request from the corresponding author. The data are not yet publicly available at the time of writing but they will.
